# Molecular pain markers correlate with pH-sensitive MRI signal in a pig model of disc degeneration

**DOI:** 10.1038/s41598-018-34582-6

**Published:** 2018-11-26

**Authors:** Maxim Bez, Zhengwei Zhou, Dmitriy Sheyn, Wafa Tawackoli, Joseph C. Giaconi, Galina Shapiro, Shiran Ben David, Zulma Gazit, Gadi Pelled, Debiao Li, Dan Gazit

**Affiliations:** 1 Skeletal Biotech Laboratory, Faculty of Dental Medicine, The Hebrew University of Jerusalem, Ein Kerem, 91120 Jerusalem Israel; 2 Department of Surgery, Cedars-Sinai Medical Center, Los Angeles, CA 90048 USA; 3 Biomedical Imaging Research Institute, Cedars-Sinai Medical Center, Los Angeles, 90048 CA USA; 4 Department of Bioengineering, University of California Los Angeles, Los Angeles, California USA; 5 Board of Governors Regenerative Medicine Institute, Cedars-Sinai Medical Center, Los Angeles, CA 90048 USA; 6 Department of Biomedical Sciences, Cedars-Sinai Medical Center, Los Angeles, CA 90048 USA; 7 Department of Imaging, Cedars-Sinai Medical Center, Los Angeles, CA 90048 USA; 8 Department of Orthopedics, Cedars-Sinai Medical Center, Los Angeles, CA 90048 USA

## Abstract

Intervertebral disc (IVD) degeneration is a leading cause of chronic low back pain that affects millions of people every year. Yet identification of the specific IVD causing this pain is based on qualitative visual interpretation rather than objective findings. One possible approach to diagnosing pain-associated IVD could be to identify acidic IVDs, as decreased pH within an IVD has been postulated to mediate discogenic pain. We hypothesized that quantitative chemical exchange saturation transfer (qCEST) MRI could detect pH changes in IVDs, and thence be used to diagnose pathologically painful IVDs objectively and noninvasively. To test this hypothesis, a surgical model of IVD degeneration in Yucatan minipigs was used. Direct measurement of pH inside the degenerated IVDs revealed a significant drop in pH after degeneration, which correlated with a significant increase in the qCEST signal. Gene analysis of harvested degenerated IVDs revealed significant upregulation of pain-, nerve- and inflammatory-related markers after IVD degeneration. A strong positive correlation was observed between the expression of pain markers and the increase in the qCEST signal. Collectively, these findings suggest that this approach might be used to identify which IVD is causing low back pain, thereby providing valuable guidance for pain and surgical management.

## Introduction

Low back pain is one of the most frequent causes of morbidity and disability^1^. It occurs most often in humans between 30 and 50 years of age due to the aging process, but also happens as a result of genetic predisposition, mechanical injuries, and a sedentary life style^2,3^. Americans spend at least 50 billion dollars each year on low back pain, the most common cause of job-related disability and a leading contributor to missed work^4^. Intervertebral disc (IVD) degeneration is believed to be a major source of chronic low back pain, and more than 90% of surgical spine procedures are performed with the aim of relieving the consequences of this degenerative process^5^. Current diagnostic approaches include computerized tomography (CT) and magnetic resonance imaging (MRI), which provide detailed soft tissue imagery but thus far fail to differentiate between a pathologically painful disc and a physiologically aging disc that does not generate pain^6,7^. Due to the shortcomings of these imaging studies, new diagnostic methods that rely on other biomarkers are being developed for low back pain.

In adults, the IVD is avascular; its nutrition depends on diffusion via the annulus fibrosus and adjacent vertebral endplates^8^. Typically, nucleus pulposus cells obtain energy by anaerobic glycolysis, leading to the production of lactic acid, which is then expelled from the IVD by diffusion. In IVDs that have degenerated due to changes in blood supply in the region, sclerosis of the subchondral bone, or endplate calcification, the supply of glucose and clearance of lactate decreases, thereby leading to a sharp drop in pH^9,10^. The pH of human IVDs ranges from 7.1 in healthy IVDs to lower than 6 in degenerated IVDs^11,12^. Therefore, a recent hypothesis was made that low pH values within the degenerated IVDs are the cause of discogenic low back pain^13,14^.

Recent advances in MRI technologies have allowed researchers to noninvasively assess pH changes in the body. Of particular note, chemical exchange saturation transfer (CEST) has been studied to measure pH-dependent MRI signal changes^15–18^. This technique exploits the chemical exchange, which is pH sensitive, between water protons and solute protons in certain molecules. Previous studies have applied CEST to detect pH changes in IVDs in pigs^19^ and humans^20^. However, the CEST signal can be affected by other contributing factors, including water relaxation parameters and solute concentration^21^. To eliminate these confounding factors, quantitative CEST (qCEST) was developed to measure the exchange rate, independently from T_1_ and T_2_ signals as well as from the solute concentration^22–25^. In the nucleus pulposus, glycosaminoglycans’ (GAGs’) hydroxyl protons can serve as an endogenous agent for qCEST to measure the pH-sensitive exchange rate with water protons. It has recently been shown that the exchange rate measured using qCEST is closely correlated to pH values found in the IVD^26^.

In this study, we hypothesized that discogenic pain is caused by a drop in pH within the degenerating IVD and thus can be detected using qCEST in a clinically relevant, large animal model of IVD degeneration. Changes in the expression profile of several pain markers, as they relate to degenerative processes and changes in pH within the IVD, were also examined *in vivo*.

## Results

### Induction of IVD degeneration

**Figure 1:**
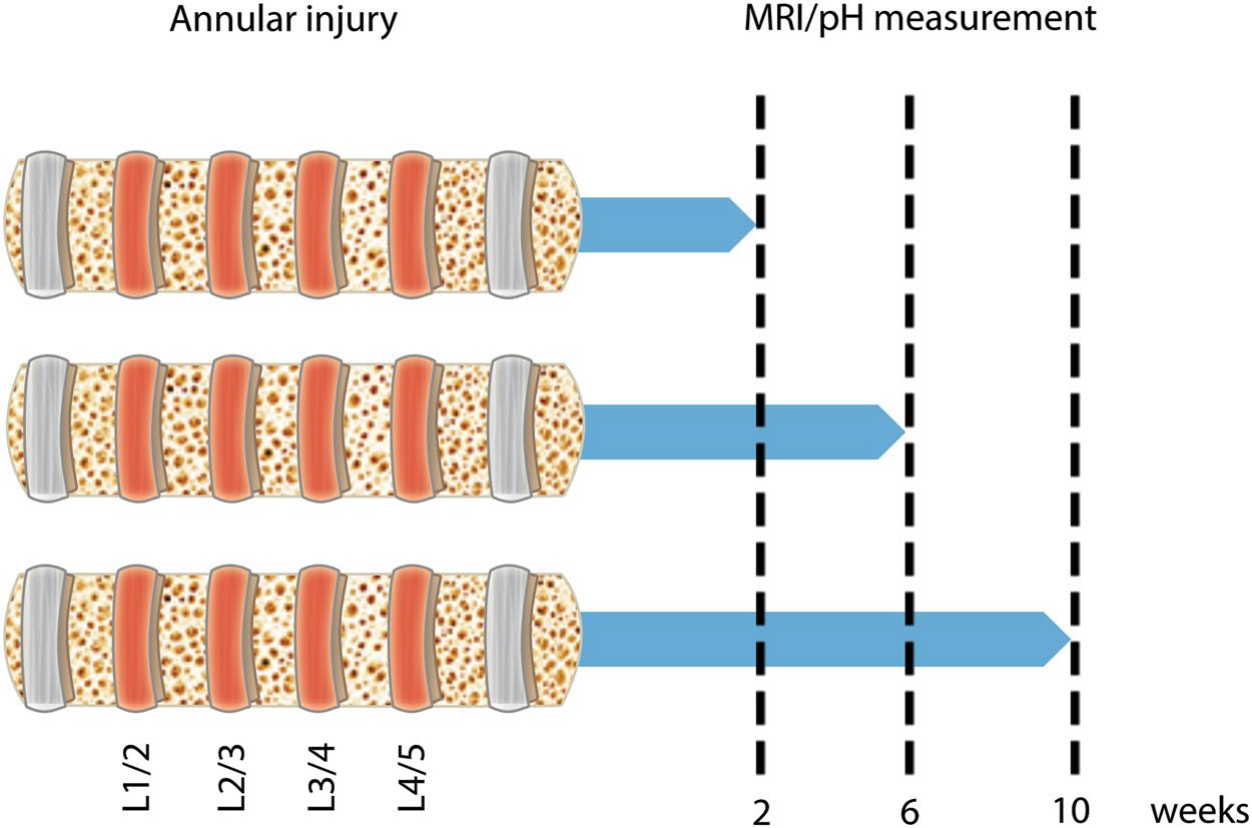
IVD degeneration timeline. Annular injuries were created in four IVD levels in minipigs to induce disc degeneration (depicted in red). Following this procedure, the animals were randomly divided into 3 groups (3 animals in each group) and imaged at 2, 6, and 10 weeks. At each time point, the animals in one group were sacrificed and the pH within their injured IVDs was measured. The IVDs were harvested for gene expression, histological, and immunohistofluoresence analyses.

**Figure 2:**
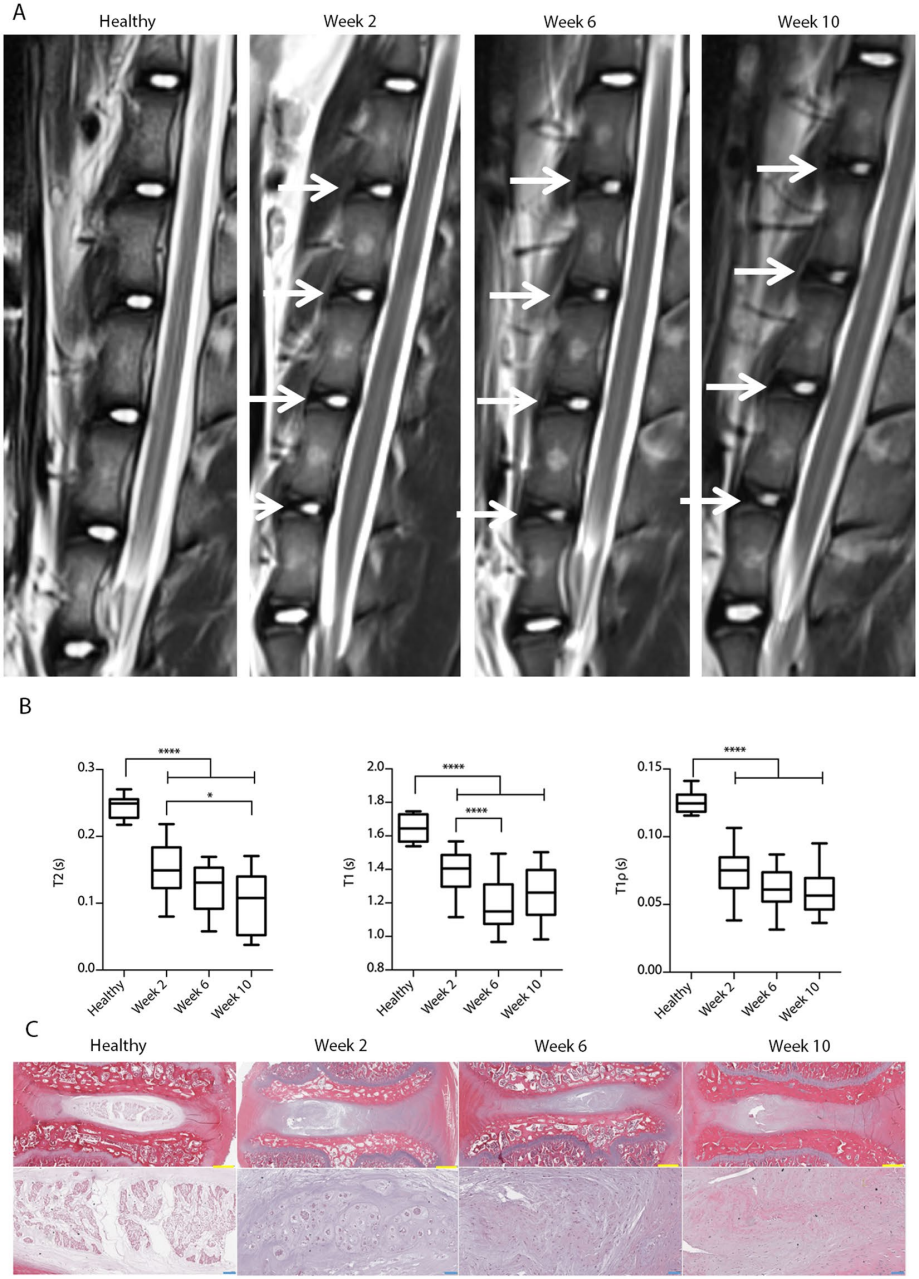
IVD degeneration following intradiscal puncture. (**A**) The progress of IVD degeneration at 2, 6, and 10 weeks following intradiscal puncture, as monitored by T_2_-weighted sagittal MRI. White arrows denote the degenerated IVDs. Quantification of (**B**) T_2_, (**C**) T_1_, and (**D**) T_1ρ_ mappings of degenerated IVDs compared to healthy controls at 2, 6, and 10 weeks after puncture (n = 12 per experimental group; *p < 0.05, ****p < 0.0001). (**E**) Hematoxylin and eosin staining of representative degenerated IVDs 2, 6, and 10 weeks after intradiscal puncture; microphotographs are shown at both low (upper subfigures; scale bars = 1 mm) and high (lower subfigures; scale bars = 100 μm) magnifications.

Minipigs underwent surgery during which the annulus fibrosus of four lumbar IVDs (L1/2, L2/3, L3/4, L4/5) was punctured to induce disc degeneration (Fig. 1). The progress of IVD degeneration was then monitored using a clinical 3T MRI system (Fig. 2A). T_2_ values following injury were significantly reduced at all time points compared to healthy porcine IVDs, suggesting loss of water content (p < 0.0001; Fig. 2B). The T_2_ values were further reduced 10 weeks after IVD puncture compared to Week 2 (p < 0.05). In addition, a significant reduction in the T_1_ signal from 1.6 to 1.4 was noticeable 2 weeks after puncture compared to the signal in healthy controls (p < 0.0001; Fig. 2C). A further reduction in the T_1_ signal to 1.2 was measured 6 weeks after IVD puncture (p < 0.0001). T_1ρ_ mapping revealed a two-fold reduction as early as 2 weeks after injury (p < 0.0001; Fig. 2D). Overall, these quantitative MR signals revealed a rapidly progressive degenerative status of the punctured IVDs^27,28^. Histology (Fig. 2E) and microphotography (Fig. S1) revealed an abnormal IVD structure and extracellular matrix following puncture of the annulus fibrosus, with extensive fibrosis and formation of cell clusters in the nucleus pulposus, which is typical of degenerated IVDs^29^.

### MR signal correlates with intradiscal pH in degenerated IVDs

**Figure 3:**
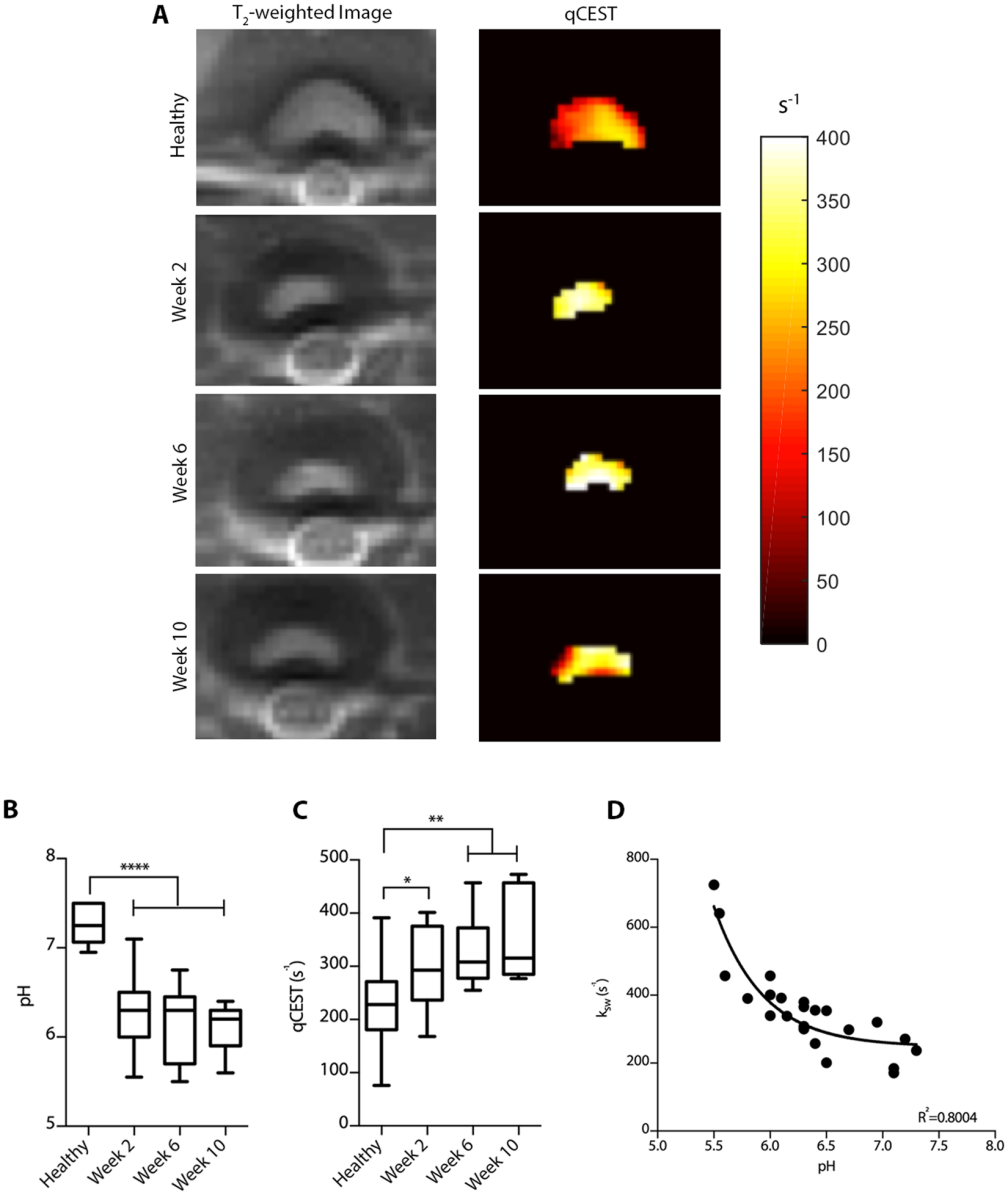
pH and qCEST changes following IVD degeneration. (**A**) Representative axial anatomical images of IVDs and their corresponding qCEST heat maps. (**B**) pH and (**C**) qCEST measurements within the degenerating IVDs at 2, 6, and 10 weeks after intradiscal puncture. (**D**) Correlation between the qCEST signal, represented by the exchange rate between the solute pool and the water pool (k_sw_), and the pH measured within the IVD following animal sacrifice (n = 12 per experimental group; *p < 0.05, **p < 0.01, ****p = 0.0001; qCEST = quantitative chemical exchange saturation transfer).

In addition to characterizing the degenerative status of IVDs based on T_2_, T_1_, and T_1ρ_ signals, we acquired qCEST signals (represented as the exchange rate *k*_*sw*_ between water protons and GAG protons) from the degenerated IVDs (Fig. 3A). A significant drop in pH from 7.2 to 6.3 was found in IVDs as soon as 2 weeks after injury and this drop continued until Week 10 (p = 0.0001, Fig. 3B). The qCEST signal significantly increased 2 weeks after injury (p < 0.05, Fig. 3C) and further increased at 6 and 10 weeks (p < 0.01). A strong correlation was observed between the qCEST signal and the direct pH reading across all time points (Fig. 3D, *k*_*sw*_ = 1.3 × 10^−pH+8^ + 248.2, R^2^ = 0.8004; p < 0.0001). In addition, a receiver operating characteristics (ROC) analysis indicated that qCEST measurements could be used to differentiate between healthy and degenerated IVDs with 81.3% sensitivity and 76.1% specificity (Fig. S2, area under the curve = 0.813, p = 0.0003).

### MR signal correlates with pain markers in degenerated IVDs

**Figure 4:**
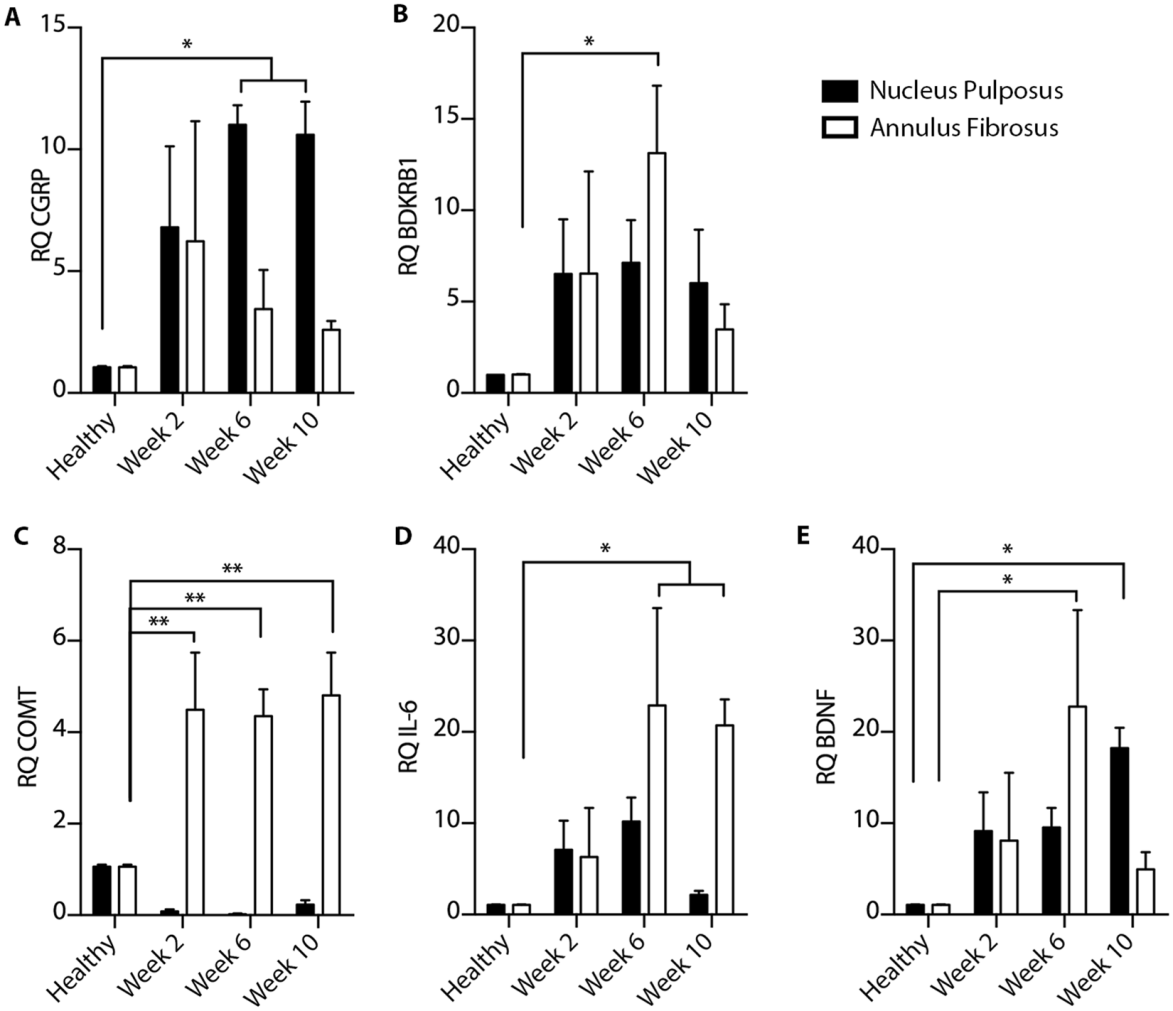
Upregulation of pain and inflammatory markers in degenerating IVDs. Quantitative RT-PCR analyses of (**A–C)** pain-related genes (CGRP, BDKRB1 and COMT) and (**D**,**E**) inflammation-related genes (IL-6 and BDNF) harvested from healthy and degenerated IVDs 2, 6, and 10 weeks after intradiscal puncture (n = 3 per group; *p < 0.05, **p < 0.01; RQ, relative quantification, CGRP, calcitonin gene-related peptide, BDKRB1, bradykinin receptor B1, COMT, catechol-*Ο*-methyltransferase, BDNF, brain-derived neurotrophic factor).

Next, we evaluated the expression of pain-related genes in the degenerated IVDs. Specifically, we evaluated the expression of calcitonin gene-related peptide (*CGRP*), bradykinin receptor B1 (*BDKRB*1), and catechol-*O*-methyltransferase (*COMT*). The earliest gene to display significant upregulation was *COMT* at Week 2 at the annulus fibrosus of the degenerated IVD (p < 0.01, Fig. 4C). By Week 6, there was a significant upregulation of all pain markers (BDKRB1 and COMT at the annulus fibrosus, CGRP at the nucleus pulposus. p < 0.05, Fig. 4A–C). Finally, in Week 10 only *CGRP* expression was significantly enhanced (p < 0.05, Fig. 4A). The inflammation marker *IL-6* was increased 20-fold at 6 and 10 weeks after IVD puncture at the annulus fibrosus (p < 0.05, Fig. 4D). In addition, brain-derived neurotrophic factor (*BDNF*), which is involved with nerve growth, was only significantly upregulated at 6 weeks after injury in the annulus fibrosus and at 10 weeks in the nucleus pulposus (p < 0.05, Fig. 4E).

**Figure 5:**
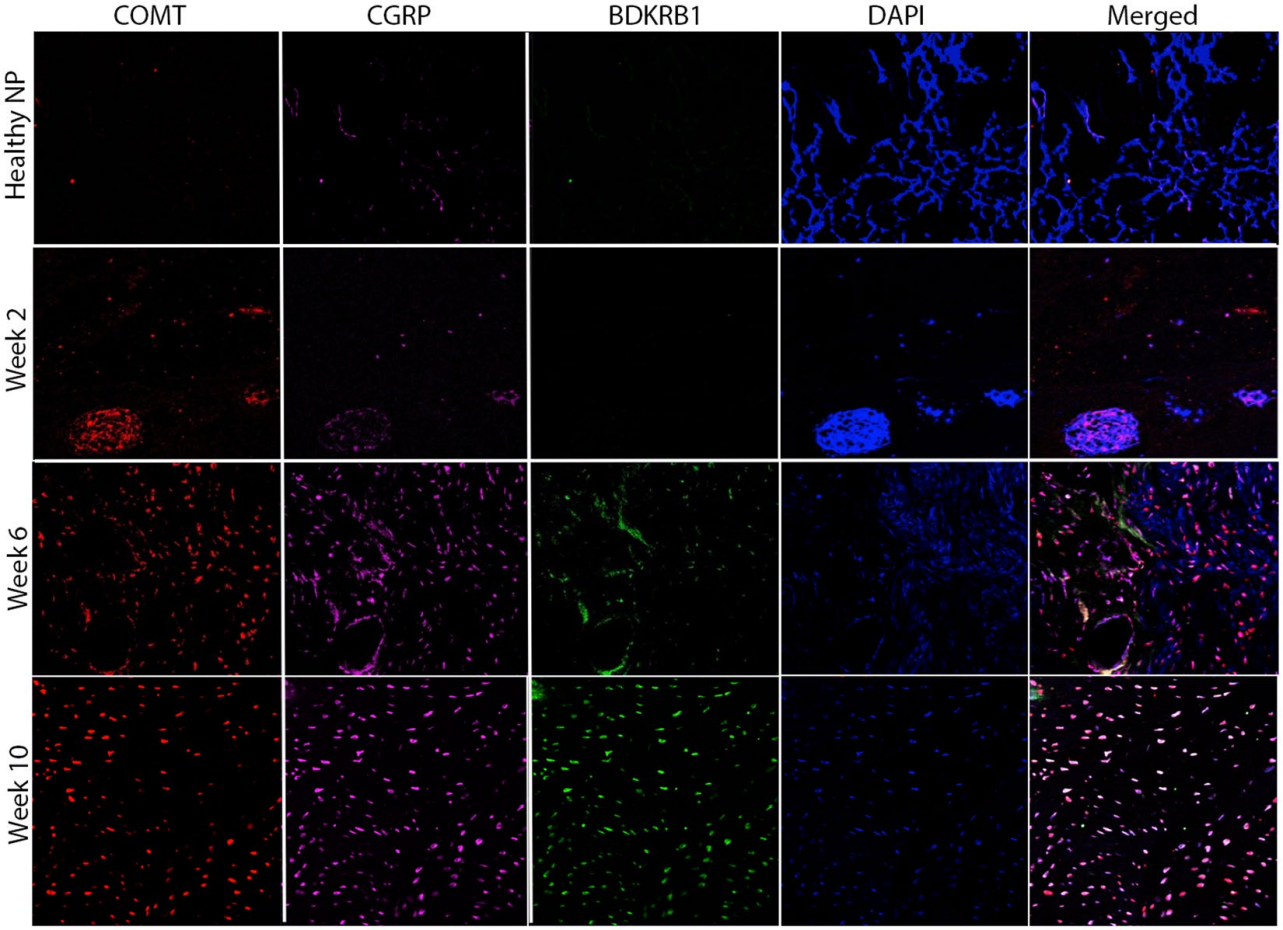
Immunofluorescence of pain-related marker upregulation. Microphotographs showing IVD tissue samples after immunostaining against COMT, CGRP, and BDKRB1, and counterstaining with DAPI; the samples were examined 2, 6, and 10 weeks after intradiscal puncture. Merged panels of the various stains are presented in the right column (NP = nucleus pulposus, CGRP = calcitonin gene-related peptide, BDKRB1 = bradykinin receptor B1, COMT = catechol-*O*-methyltransferase).

**Figure 6:**
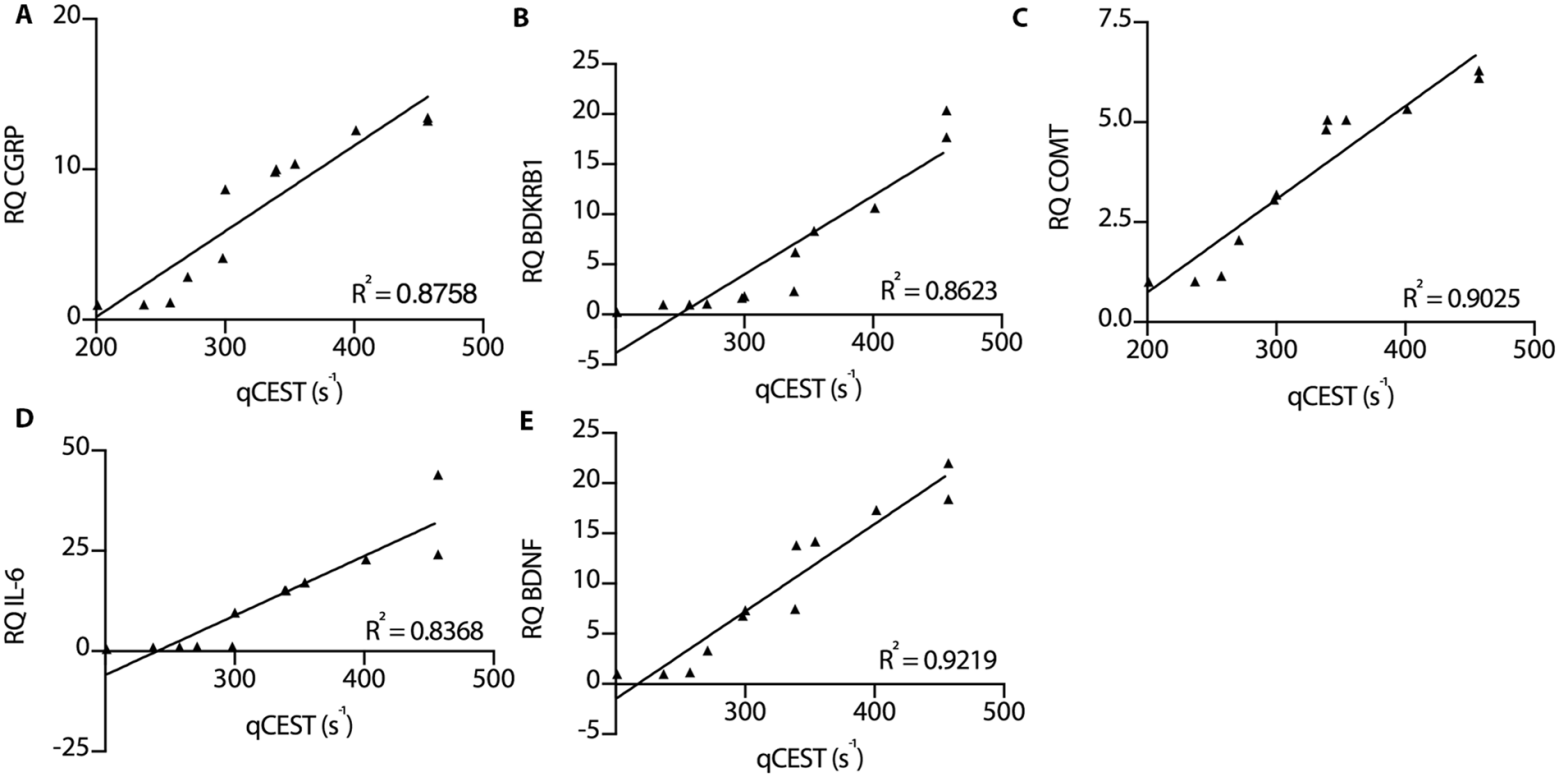
Linear correlation between qCEST and biomarkers in degenerating IVDs. Correlation plots between qCEST signal and expression levels of (**A**) CGRP, (**B**) BDKRB1, (**C**) COMT, (**D**) IL-6, and (**E**) BDNF extracted from degenerated and healthy annulus fibrosus and nucleus pulposus (RQ, relative quantification).

Immunofluorescent staining of the nucleus pulposus revealed increased protein expression and colocalization of pain markers in degenerated IVDs compared to healthy IVDs (Fig. 5). Co-localization of COMT and IL-6 was observed within the degenerated IVDs (Fig. S3). Finally, strong linear correlations between qCEST signals and expression levels from both the nucleus pulposus and annulus fibrosus of CGRP (R^2^ = 0.8758, p < 0.0001), BDKRB1 (R^2^ = 0.8623, p < 0.0001), COMT (R^2^ = 0.9025, p < 0.0001), IL-6 (R^2^ = 0.8368, p < 0.0001), and BDNF (R^2^ = 0.9219, p < 0.0001) were observed (Fig. 6). Combined, these results show that the qCEST signal is anti-correlated with pH and correlated with pain and inflammatory markers within the IVDs.

## Discussion

In this study, we used MRI to detect intradiscal pH changes that correlated with upregulation of pain markers in a minipig model of IVD degeneration. We showed that disc degeneration was achieved by 10 weeks following IVD puncture, as detected by MRI and histology. A significant drop in pH was observed during the degenerative process as well as a significant increase in the qCEST signal. These changes were detected as early as 2 weeks after injury. The qCEST signals were anti-correlated with pH measurements obtained directly from the degenerated IVDs. Gene analysis revealed upregulation of pain markers in degenerated IVDs—a finding that was strongly correlated with the qCEST signal at various time points.

Quantitative MR sequences offer new objective markers capable of differentiating healthy and injured IVDs. Conventional quantitative protocols of T_1_- and T_2_-relaxation times, and more recently T_1ρ_ mapping, are well established in the literature as proxies for the detection of pathological changes within IVDs^6,30–33^. Of particular interest, the T_1ρ_ signal has been compared to findings of lumbar provocative discography as a quantitative biomarker of low back pain^34^. In that study, researchers demonstrated that loss of proteoglycans, as shown by a reduced T_1ρ_ signal, can be used to detect painful IVDs with a moderate correlation to discography. However, these results most likely stem from the relatively low specificity of the method, as the loss of proteoglycans is a common feature of IVD degeneration and does not necessarily correlate to pain symptoms^35^. In addition, performing the discography procedure immediately before MRI raises the question of whether the injection of a contrast agent affects the measured T_1ρ_ signal within the IVD due to increased water content.

Newer methods in which gagCEST protocols are used to evaluate GAG content have also been tested to detect low back pain. Wada *et al*. showed that there is a correlation between the gagCEST signal and the Pfirrmann score in patients with low back pain (r = −0.675)^36^. A similar work, in which the same approach was used, showed a weaker correlation in patients (r = −0.449)^37^. While no direct correlation to pain scoring was shown in either work, the gagCEST signal depends on the T_1_ and T_2_ signals, and the collagen content, making this approach less reliable^20,21,38^. Although loss of GAGs occurs early in the degenerative process of IVDs and can therefore be used as an early marker of IVD degeneration^35^, no causative relationship has been shown between depletion of GAGs and discogenic low back pain.

In addition to GAG loss, disc acidification has been implicated in discogenic pain development. This process could irritate locally infiltrating nerve fibers, thereby triggering discogenic pain. Several publications have shown that cells isolated from degenerated IVDs overexpress and secrete several pain-related and neurotrophic factors that enhance nerve growth, including BDNF and nerve growth factor^39–41^. In addition, conditioned medium from degenerate IVD cells promote increased neurite outgrowth in nerve cells^39^. There is evidence that in degenerative IVDs, the number of nerve fibers at the outer layer of the annulus fibrosus increases and nerve fibers grow into inner portions of the annulus and, occasionally, even into the nucleus pulposus—areas that otherwise are not innervated in healthy individuals^42–44^.

In the pig disc degeneration model, we studied changes in gene expression of pain and inflammatory markers both in the nucleus pulposus and the annulus fibrosus. Our findings showed that pain markers were upregulated within the annulus fibrosus as soon as 2 weeks after injury, followed by pain marker upregulation at both locations. We also observed overexpression of BDNF in the annulus fibrosus 6 weeks after the injury; this was followed by expression of BDNF in the nucleus pulposus at 10 weeks. These patterns of expression concur with reports of nerve fiber penetration into the nucleus pulposus. A recent study showed that acidic pH directly causes an increase in several pro-inflammatory, neurotrophic, and pain-related factors in cultured human nucleus pulposus cells, including IL-6 and BDNF, in a similar fashion to our findings^45^. This upregulation may be responsible for the ingrowth of nerve fibers into degenerate IVDs, and low pH is a contributing factor to this process.

Furthermore, acid-sensing ion channels (ASICs), extracellular receptors that respond to a low pH, were found to be upregulated after exposure to an acidic environment in nucleus pulposus cells and to regulate the biological activity of these cells^46–48^. ASIC3 was found to protect IVD cells from apoptosis in low pH conditions^48^. In the same study, it was shown that nerve growth factor is required to maintain a basal level of ASIC3; thus, it is conceivable that IVD cells secrete nerve growth factor to protect themselves from an acidic environment within the IVD and indirectly activate pain fibers in this process. Researchers have inhibited the activity of ASICs in nucleus pulposus cells, showing a reduction in the expression of pro-inflammatory, neurotrophic, and pain-related factors in an acidic environment^45,49^. Although the exact mechanism underlying these processes remains elusive, these findings indicate a close relationship between low pH and discogenic pain.

One of the biggest challenges in using animals to study IVD degeneration relates to pain perception. Gruber *et al*. conducted a genome-wide analysis of IVD specimens from human patients to examine key pain-related genes associated with discogenic pain^50^. Similar to our findings, these researchers’ work showed upregulation of CGRP, COMT, and BDKRB1 in pathological IVD specimens; this was confirmed by positive immunohistochemical staining in the annulus. These pain-related factors could mediate hyperalgesia in chronic inflammation^51^, stimulate sensory nerves in inflamed tissues^52^, and participate in the metabolism of pain neurotransmitters, including noradrenalin, adrenaline, and dopamine^53^. CGRP upregulation was also observed in an acute injury model of the IVD in a neurotrophic gene expression–dependent manner^54^. Thus, our findings could serve as a biological clue that our degeneration model causes discogenic pain in experimental animals.

Other previous studies attempted to evaluate pH as a biomarker for diagnosing discogenic low back pain. Zuo *et al*. demonstrated the feasibility of acquiring localized ^1^H spectra using a 3.0T imager in intact bovine and human cadaveric IVDs to quantify lactate causing low pH^55^. Translating this technique to *in vivo* spectroscopy suffers from several limitations. As stated by the authors, it is difficult to differentiate lactate from lipid peaks because their resonance frequencies are close. Another limitation is inadequate quantification of metabolites in IVDs within the collapsed space. A later study from the same group characterized IVDs *in vivo* by using magnetic resonance spectroscopy (MRS)^56^. A significantly elevated water/proteoglycan area ratio was found in IVDs identified as the source of pain by discography. *In vivo* MRS is challenging because of a low signal-to-noise ratio, physiological motion, and bone susceptibility–induced line broadening, making the assessment of lactate imprecise. Another study found a nonlinear dependence of the CEST effect of GAG on pH in porcine IVD specimens^19^. However, that study was performed *ex vivo* using a 7.0T MRI unit, which currently is not widely available in the clinical setting.

While we observed the animals for up to 10 weeks after IVD puncture, the subclinical degenerative processes in humans can take many years. In this study, we observed in all injured IVDs a drop in pH with upregulation of several pain markers, which persisted for 10 weeks. A long-term study will probably be required to broaden the validity of this approach to include chronic IVD degeneration and differentiate between degenerated painful discs and degenerated nonpainful discs in humans.

Overall, these results show great promise for future human studies, as this approach can be easily and readily implemented using clinical MRI systems. qCEST protocols can be used to provide additional information on IVD physiology and to detect pH changes associated with early IVD degeneration, thus providing an early, noninvasive, and objective diagnosis that can be used to prompt early intervention and prevention of chronic low back pain.

## Materials and Methods

### Study design

The objective of our study was to develop a pH-level–based MRI approach to diagnose low back pain. Our prespecified hypothesis was that the acidic intradiscal environment that contributes to discogenic pain can be detected noninvasively using qCEST imaging. qCEST was investigated for its capacity to detect pH changes within IVDs and predict pain marker expression. Nine healthy female skeletally mature Yucatan minipigs (S&S Farms; average age 7 months, 35–40 kg) were included in this study. The sample size that we used was estimated to achieve a power of 0.8 and α = 0.05 using one-way ANOVA. All animals underwent a surgical procedure in which multiple-level lumbar IVD degeneration was induced (Fig. 1). The degeneration was induced in the lumbar spine as it is the major source of low back pain since it is affected the most by age-related degenerative processes^57^. Afterward, the animals underwent MRI at 2, 6, and 10 weeks. At each time-point, three minipigs were randomly selected for euthanasia to directly measure the pH within the affected IVD as well as to analyze gene expression, histological architecture, and protein expression. IVD degeneration was evaluated based on imaging parameters and histological findings. Pain was detected based on gene expression and findings of immunofluorescence, and was compared to qCEST measurements within the IVDs. Animals that developed periprocedural complications, such as nerve damage or signs of distress, were eliminated from the study.

### IVD degeneration animal model

All experiments using animals and procedures of animal care and handling were carried out in strict accordance with the recommendations of The Association for Assessment and Accreditation of Laboratory Animal Care. All animal procedures were approved by the Cedars-Sinai Medical Center institutional review board for animal experiments. A previously published IVD degeneration model^58^ was adapted for this study. Briefly, following an 18-hr preoperative fast, each minipig was sedated using intramuscular acepromazine (0.25 mg/kg), ketamine (20 mg/kg), and atropine (0.02–0.05 mg/kg). The animal was then administered propofol (2 mg/kg) intravenously and endotracheal intubation was performed. Anesthesia was maintained using 1–3.5% inhaled isoflurane for the duration of the procedure. IVD degeneration was induced by a single annular injury as published elsewhere^59^. Specifically, under fluoroscopic guidance, a 14-G VertePort needle (Stryker, Kalamazoo, MI) was used to penetrate and injure the annulus fibrosus of the IVD parallel to the endplate via a posterolateral approach. This procedure was repeated at four target levels: L1/L2, L2/L3, L3/L4, and L4/L5.

### *In vivo* MRI

Imaging studies were performed using a 3T clinical MRI system (Magnetom Verio; Siemens Healthcare, Erlangen, Germany). Animals were placed in the right decubitus position with body array coils centered on lumbar spinous processes. CEST MRI, T1, T2 and T_1ρ_ mapping were performed in the axial plane for each IVD. Total scan time was approximately 40 minutes for each IVD.

CEST MRI was performed using a two-dimensional reduced field of view TSE CEST sequence (TR/TE = 10500/10 ms, 2 averages, single shot)^60^. For each IVD, images were acquired in the axial plane with a slice thickness of 3 mm, a field of view of 140 × 40 mm^2^, and a spatial resolution of 1.1 × 1.1 mm^2^. The CEST saturation module consisted of 39 Gaussian-shaped pulses, with a duration t_p_ = 80 ms for each pulse and an interpulse delay t_d_ = 80 ms (duty cycle = 50%, total saturation duration T_s_ = 6240 ms) at saturation flip angles of 900, 1500, 2100, and 3000 [B_1_ amplitudes = flip angle/(gt_p_) = 0.73, 1.22, 1.71, and 2.45 µT]; the Z-spectrum was acquired with 10 different saturation frequencies at ±1.6, ±1.3, ±1.0, ±0.7, and ±0.4 ppm.

The B_0_ field was corrected using a water saturation shift referencing (WASSR) map^61^ with the same imaging readout parameters. The CEST saturation module for WASSR map consisted of 2 Gaussian-shaped pulses, with a duration t_p_ = 30 ms for each pulse and an interpulse delay t_d_ = 30 ms (duty cycle = 50%, total saturation duration T_s_ = 120 ms) at saturation flip angles of 90°. The images were acquired from −0.8 ppm to 0.8 ppm with a step size of 0.2 ppm.

T_1_ mapping was performed using an inversion recovery TSE sequence with seven different inversion times (TI = 50, 150, 350, 700, 1050, 1400, and 2000 ms), TR/TE = 6000/12 ms, FOV = 280 × 280 mm^2^, and spatial resolution = 1.1 × 1.1 × 3 mm^3^.

T_2_ mapping was performed using a TSE sequence with various echo delays (TE = 12, 25, 50, 99, 199, and 397 ms; TR = 6000 ms), FOV = 280 × 280 mm^2^, and spatial resolution = 1.1 × 1.1 × 3 mm^3^.

T_1ρ_ mapping was performed using an rFOV TSE sequence with various spin lock times (TSL = 0, 10, 40, and 80 ms). A spin-lock frequency of 300 Hz was used with TR/TE = 3500/9.1 ms, 1 average, FOV = 140 × 40 mm^2^, and spatial resolution = 1.1 × 1.1 × 3 mm^3^.

### MRI data analysis

Imaging data analysis was performed with custom-written programs in MATLAB (MathWorks, Natick, Massachusetts, USA) as previously reported^26^.

The inverse CEST difference (CESTR_ind_) was calculated for each B_1_ amplitude using the following equation. Z_+1.0 ppm_ and Z_−1.0 ppm_ are the normalized signal intensity acquired at +1.0 ppm and −1.0 ppm after B_0_ correction respectively.

$$\frac{1}{{{\rm{CESTR}}}_{{\rm{ind}}}}=\frac{1}{\frac{1}{{{\rm{Z}}}_{+1.0{\rm{ppm}}}}-\frac{1}{{{\rm{Z}}}_{-1.0{\rm{ppm}}}}}$$

As shown in the previous study^26^, 1/CESTR_ind_ can be described as a linear function of $1/{\omega }_{1}^{2}$.

$$\frac{1}{{{\rm{CESTR}}}_{{\rm{ind}}}}\approx \frac{{R}_{1w}}{{\rm{DC}}\cdot {f}_{r}\cdot {k}_{sw}\cdot {c}_{1}}+\frac{{k}_{sw}\cdot ({R}_{2s}+{k}_{sw})\cdot {R}_{1w}\cdot {c}_{2}^{2}}{{\rm{DC}}\cdot {f}_{r}\cdot {k}_{sw}\cdot {c}_{1}}\frac{1}{{\omega }_{1}^{2}}$$

where DC stands for duty cycle; *c*_*1*_ and *c*_2_ describe the shape of Gaussian saturation pulses $$({c}_{1}=\sigma \sqrt{2\pi }/{t}_{p},\,$$$$\,{c}_{2}={c}_{1}\sqrt{\sqrt{2}};$$ *σ* and *t*_*p*_ are the width and length of the Gaussian pulse). *ω*_1_ is defined as flip angle/pulse duration (in this study, 0.73, 1.22, 1.71, and 2.45 µT).

Linear regression was performed between 1/CESTR_ind_ and $1/{\omega }_{1}^{2}$ to estimate the slope *m* and intercut *n*, and eventually estimate *k*_*sw*_.

$${k}_{sw}=\frac{\sqrt{{{\rm{R}}}_{2{\rm{s}}}^{2}+\frac{4m}{n\cdot {c}_{2}^{2}}}-{{\rm{R}}}_{2{\rm{s}}}}{2}$$

The T_1_ maps were obtained by pixel-by-pixel lease-squares fitting of the signal equation *I* = *I*_0_[1 − (1 + *η*) · exp (−TI/*T*_1_)] where *I* is the signal intensity, TI is the inversion time and *η* is the inversion efficiency. The T_2_ maps were obtained by fitting the signal equation *I* = *I*_0_ · exp (−TI/*T*_2_) where *I* is the signal intensity and TE is the echo time. The T_1ρ_ maps were obtained by fitting the signal equation *I* = *I*_0_ · exp (−TSL/*T*_1*ρ*_) where *I* is the signal intensity and TSL is the spin lock time.

### IVD pH measurement

Intradiscal pH measurements were made immediately after animal sacrifice. A single pH value was obtained for each IVD. The spine was surgically exposed, and a custom-made needle-shaped tissue pH probe (Warner Instruments, Hamden, Connecticut, USA; Fig. S4) was inserted into the nucleus pulposus following a thin incision in the annulus fibrosus.

### Gene expression analysis

Quantitative RT-PCR was performed on RNA extracted from degenerated IVDs harvested at 2, 6, and 10 weeks after induction of’ IVD degeneration and compared to control healthy discs. The expression of genes from degenerative IVDs was compared to healthy IVDs harvested from each time point. Total RNA was extracted from the annulus fibrosus and the nucleus pulposus by using the RNeasy Mini kit (Qiagen GmbH, Hilden, Germany) according to the manufacturer’s protocol. RNA was retrotranscribed using random primers and reverse transcriptase (Promega Corp., Madison, WI, USA). Quantitative real-time PCR was performed using an ABI 7500 Prism system (Applied Biosystems, Foster City, CA). The expression of the following porcine genes was quantified: Bradykinin receptor B1 (BDKRB1; Ss03389804_s1, Thermo Fisher Scientific, Waltham, MA, USA), calcitonin gene–related peptide (CGRP; Ss03386432_uH) and catechol-*O*-methyltransferase (COMT; Ss04247881_g1), interleukin-6 (IL-6; Ss03384604_u1), and brain-derived neurotrophic factor (BDNF; Ss03822335_s1). Porcine 18 s was used as a housekeeping gene control.

### Histological analysis and immunofluorescence imaging

Histological analysis was performed on degenerated IVDs harvested 2, 6, and 10 weeks after IVD puncture. The IVDs were sectioned and stained using hematoxylin and eosin, as previously described^62^. For immunofluorescent staining, the IVD tissues were deparaffinized, and the antigens were retrieved by incubation in preheated Target Retrieval Solution (Dako, Carpinteria, CA) for 45 min in 37 °C. Nonspecific antigens were blocked by applying blocking serum–free solution (Dako). Slides were stained with primary antibodies against BDKRB1, CGRP, COMT, IL-6, and BDNF (Table S1). The primary antibodies were applied to the slides, which were then incubated in 4 °C overnight. Then these antibodies were washed off using PBS, and the slides were incubated with a secondary antibody for 1 hr at room temperature, after which it also was washed off with PBS. Slides were then stained with 4′,6-diamidino-2-phenylindole dihydrochloride (DAPI, 1 μg/ml) for 5 min in the dark, after which they were washed three times with PBS. A VectaMount mounting medium (Vector Laboratories, Burlingame, CA) was applied to the tissue. The slides were imaged using a 4-channel Laser Scanning Microscope 780 (Zeiss, Pleasanton, CA) with ×20 magnification, z-stacking, and 5 × 5 tile scanning. For zoom-in images, a single z-stacked image was generated. All samples were scanned using the same gain and exposure settings.

### Statistical analysis

GraphPad Prism 5.0 f software (GraphPad Prism, San Diego, CA) was used to analyze the data. Data analysis was conducted using one-way or two-way ANOVA with Tukey’s multiple comparison post hoc test. Results are presented as means ± SE. In box-and-whisker diagrams, the median is shown with a horizontal line, the box extends from the 25th to the 75th percentile, and the whiskers extend from the smallest value to the largest. Pearson correlation was performed between qCEST, pH, and RT-PCR values. ROC curve that depicts the diagnostic ability of qCEST to detect a true positive event of low back pain was generated for this study and the area under the curve was calculated. P values that were less than 0.05 were considered to be statistically significant.

## Electronic supplementary material

Supplementary Information

## Data Availability

All materials are available from commercial sources or can be derived using methods described in this study. All relevant data are reported in the article.

## References

[1] Andersson GB (1999). Epidemiological features of chronic low-back pain. Lancet.

[2] Knox J (2011). The incidence of low back pain in active duty United States military service members. Spine (Phila Pa 1976).

[3] Lefevre-Colau MM (2009). Frequency and interrelations of risk factors for chronic low back pain in a primary care setting. PLoS One.

[4] Asicioglu O, Gungorduk K, Yildirim G, Aslan H, Gunay T (2014). Maternal and perinatal outcomes of eclampsia with and without HELLP syndrome in a teaching hospital in western Turkey. J Obstet Gynaecol.

[5] An HS (2004). Introduction: disc degeneration: summary. Spine (Phila Pa 1976).

[6] Majumdar Sharmila, Link Thomas M., Steinbach Lynne S., Hu Serena, Kurhanewicz John (2011). Diagnostic Tools and Imaging Methods in Intervertebral Disk Degeneration. Orthopedic Clinics of North America.

[7] Kang CH (2009). Can magnetic resonance imaging accurately predict concordant pain provocation during provocative disc injection?. Skeletal Radiol.

[8] Raj PP (2008). Intervertebral disc: anatomy-physiology-pathophysiology-treatment. Pain Pract.

[9] Menkin V (1960). Biochemical Mechanisms in Inflammation. Br Med J.

[10] Urban JP, Smith S, Fairbank JC (2004). Nutrition of the intervertebral disc. Spine.

[11] Ichimura K, Tsuji H, Matsui H, Makiyama N (1991). Cell culture of the intervertebral disc of rats: factors influencing culture, proteoglycan, collagen, and deoxyribonucleic acid synthesis. J Spinal Disord.

[12] Nachemson A (1969). Intradiscal measurements of pH in patients with lumbar rhizopathies. Acta Orthop Scand.

[13] Melkus, G., Grabau, M., Karampinos, D. C. & Majumdar, S. *Ex vivo* porcine model to measure pH dependence of chemical exchange saturation transfer effect of glycosaminoglycan in the intervertebral disc. *Magnetic resonance in medicine: official journal of the Society of Magnetic Resonance in Medicine/Society of Magnetic Resonance in Medicine* (2013). 10.1002/mrm.24838 PMC3883956 23818244

[14] Liang CZ (2012). The relationship between low pH in intervertebral discs and low back pain: a systematic review. Arch Med Sci.

[15] Zhou J, Payen JF, Wilson DA, Traystman RJ, van Zijl PC (2003). Using the amide proton signals of intracellular proteins and peptides to detect pH effects in MRI. Nat Med.

[16] Sun PZ (2008). Relaxation-compensated fast multislice amide proton transfer (APT) imaging of acute ischemic stroke. Magn Reson Med.

[17] Sun PZ, Zhou J, Sun W, Huang J, van Zijl PC (2007). Detection of the ischemic penumbra using pH-weighted MRI. J Cereb Blood Flow Metab.

[18] Zhou J, van Zijl PC (2011). Defining an Acidosis-Based Ischemic Penumbra from pH-Weighted MRI. Transl Stroke Res.

[19] Melkus G, Grabau M, Karampinos DC, Majumdar S (2014). *Ex vivo* porcine model to measure pH dependence of chemical exchange saturation transfer effect of glycosaminoglycan in the intervertebral disc. Magn Reson Med.

[20] Liu Q (2015). Detection of low back pain using pH level-dependent imaging of the intervertebral disc using the ratio of R1rho dispersion and -OH chemical exchange saturation transfer (RROC). Magn Reson Med.

[21] Vinogradov E, Sherry AD, Lenkinski RE (2013). CEST: from basic principles to applications, challenges and opportunities. J Magn Reson.

[22] Wu R, Xiao G, Zhou IY, Ran C, Sun PZ (2015). Quantitative chemical exchange saturation transfer (qCEST) MRI - omega plot analysis of RF-spillover-corrected inverse CEST ratio asymmetry for simultaneous determination of labile proton ratio and exchange rate. NMR Biomed.

[23] Sun PZ, Wang Y, Dai Z, Xiao G, Wu R (2014). Quantitative chemical exchange saturation transfer (qCEST) MRI–RF spillover effect-corrected omega plot for simultaneous determination of labile proton fraction ratio and exchange rate. Contrast Media Mol Imaging.

[24] Wu R, Longo DL, Aime S, Sun PZ (2015). Quantitative description of radiofrequency (RF) power-based ratiometric chemical exchange saturation transfer (CEST) pH imaging. NMR Biomed.

[25] Sun PZ, Xiao G, Zhou IY, Guo Y, Wu R (2016). A method for accurate pH mapping with chemical exchange saturation transfer (CEST) MRI. Contrast Media Mol Imaging.

[26] Zhou Z (2016). Quantitative chemical exchange saturation transfer MRI of intervertebral disc in a porcine model. Magn Reson Med.

[27] Trattnig S (2010). Lumbar intervertebral disc abnormalities: comparison of quantitative T2 mapping with conventional MR at 3.0 T. Eur Radiol.

[28] Auerbach JD (2006). *In vivo* quantification of human lumbar disc degeneration using T(1rho)-weighted magnetic resonance imaging. Eur Spine J.

[29] Zhao CQ, Wang LM, Jiang LS, Dai LY (2007). The cell biology of intervertebral disc aging and degeneration. Ageing Res Rev.

[30] Chatani K, Kusaka Y, Mifune T, Nishikawa H (1993). Topographic differences of 1H-NMR relaxation times (T1, T2) in the normal intervertebral disc and its relationship to water content. Spine (Phila Pa 1976).

[31] Chiu EJ (2001). Magnetic resonance imaging measurement of relaxation and water diffusion in the human lumbar intervertebral disc under compression *in vitro*. Spine (Phila Pa 1976).

[32] Boos N (1997). Tissue characterization of symptomatic and asymptomatic disc herniations by quantitative magnetic resonance imaging. J Orthop Res.

[33] Blumenkrantz G (2010). *In vivo* 3.0-tesla magnetic resonance T1rho and T2 relaxation mapping in subjects with intervertebral disc degeneration and clinical symptoms. Magn Reson Med.

[34] Borthakur A (2011). T1rho magnetic resonance imaging and discography pressure as novel biomarkers for disc degeneration and low back pain. Spine (Phila Pa 1976).

[35] Urban JP, McMullin JF (1985). Swelling pressure of the inervertebral disc: influence of proteoglycan and collagen contents. Biorheology.

[36] Wada T (2017). Glycosaminoglycan chemical exchange saturation transfer in human lumbar intervertebral discs: Effect of saturation pulse and relationship with low back pain. J Magn Reson Imaging.

[37] Haneder S (2013). Assessment of glycosaminoglycan content in intervertebral discs using chemical exchange saturation transfer at 3.0 Tesla: preliminary results in patients with low-back pain. Eur Radiol.

[38] Kim J, Wu Y, Guo Y, Zheng H, Sun PZ (2015). A review of optimization and quantification techniques for chemical exchange saturation transfer MRI toward sensitive *in vivo* imaging. Contrast Media Mol Imaging.

[39] Richardson SM (2012). Degenerate human nucleus pulposus cells promote neurite outgrowth in neural cells. PLoS One.

[40] Purmessur D, Freemont AJ, Hoyland JA (2008). Expression and regulation of neurotrophins in the nondegenerate and degenerate human intervertebral disc. Arthritis Res Ther.

[41] Navone SE (2012). Expression of neural and neurotrophic markers in nucleus pulposus cells isolated from degenerated intervertebral disc. J Orthop Res.

[42] Freemont AJ (1997). Nerve ingrowth into diseased intervertebral disc in chronic back pain. Lancet.

[43] Coppes MH, Marani E, Thomeer RT, Oudega M, Groen GJ (1990). Innervation of annulus fibrosis in low back pain. Lancet.

[44] Peng B (2005). The pathogenesis of discogenic low back pain. J Bone Joint Surg Br.

[45] Gilbert HT, Hodson N, Baird P, Richardson SM, Hoyland JA (2016). Acidic pH promotes intervertebral disc degeneration: Acid-sensing ion channel -3 as a potential therapeutic target. Sci Rep.

[46] Ohtori S (2006). Up-regulation of acid-sensing ion channel 3 in dorsal root ganglion neurons following application of nucleus pulposus on nerve root in rats. Spine (Phila Pa 1976).

[47] Cuesta A (2014). Acid-sensing ion channels in healthy and degenerated human intervertebral disc. Connect Tissue Res.

[48] Uchiyama Y (2007). Expression of acid-sensing ion channel 3 (ASIC3) in nucleus pulposus cells of the intervertebral disc is regulated by p75NTR and ERK signaling. J Bone Miner Res.

[49] Liu, J. *et al*. Biological behavior of human nucleus pulposus mesenchymal stem cells in response to changes in the acidic environment during intervertebral disc degeneration. *Stem Cells Dev* (2017). 10.1089/scd.2016.0314 28298159

[50] Gruber HE, Hoelscher GL, Ingram JA, Hanley EN (2012). Genome-wide analysis of pain-, nerve- and neurotrophin -related gene expression in the degenerating human annulus. Mol Pain.

[51] Dray A, Perkins M (1993). Bradykinin and inflammatory pain. Trends Neurosci.

[52] Donnerer J, Schuligoi R, Stein C (1992). Increased content and transport of substance P and calcitonin gene-related peptide in sensory nerves innervating inflamed tissue: evidence for a regulatory function of nerve growth factor *in vivo*. Neuroscience.

[53] Andersen S, Skorpen F (2009). Variation in the COMT gene: implications for pain perception and pain treatment. Pharmacogenomics.

[54] Orita S (2010). Inhibiting nerve growth factor or its receptors downregulates calcitonin gene-related peptide expression in rat lumbar dorsal root ganglia innervating injured intervertebral discs. J Orthop Res.

[55] Zuo J (2009). Assessment of intervertebral disc degeneration with magnetic resonance single-voxel spectroscopy. Magn Reson Med.

[56] Zuo J (2012). *In vivo* intervertebral disc characterization using magnetic resonance spectroscopy and T1rho imaging: association with discography and Oswestry Disability Index and Short Form-36 Health Survey. Spine (Phila Pa 1976).

[57] Kjaer P, Leboeuf-Yde C, Korsholm L, Sorensen JS, Bendix T (2005). Magnetic resonance imaging and low back pain in adults: a diagnostic imaging study of 40-year-old men and women. Spine (Phila Pa 1976).

[58] Mizrahi O (2013). Nucleus pulposus degeneration alters properties of resident progenitor cells. Spine J.

[59] Kim KS, Yoon ST, Li J, Park JS, Hutton WC (2005). Disc degeneration in the rabbit: a biochemical and radiological comparison between four disc injury models. Spine (Phila Pa 1976).

[60] Liu Q (2013). Reliable chemical exchange saturation transfer imaging of human lumbar intervertebral discs using reduced-field-of-view turbo spin echo at 3.0 T. NMR Biomed.

[61] Kim M, Gillen J, Landman BA, Zhou J, van Zijl PC (2009). Water saturation shift referencing (WASSR) for chemical exchange saturation transfer (CEST) experiments. Magn Reson Med.

[62] Sheyn D (2013). PTH promotes allograft integration in a calvarial bone defect. Mol Pharm.

