# Supplementary material for: Molecular pain markers correlate with pH-sensitive MRI signal in a pig model of disc degeneration
Source: Sci Rep. 2018 Nov 26;8:17363. doi: 10.1038/s41598-018-34582-6 (PMC6255799; doi:10.1038/s41598-018-34582-6)
Supplement: Supplementary file 1 — Supplementary Information [file 41598_2018_34582_MOESM1_ESM.pdf]

Title: Molecular pain markers correlate with pH-sensitive MRI signal in a pig model of disc degeneration

Authors: Maxim Bez[1,2]†, Zhengwei Zhou[3,4]†, Dmitriy Sheyn[2,5], Wafa Tawackoli[2,3,5,6], Joseph C. Giaconi[7], Galina Shapiro[1], Shiran Ben David[2,5], Zulma Gazit[1,2,5,8], Gadi Pelled[1,2,5,6,8], Debiao Li[3,4,7], Dan Gazit[1,2,3,5,6,8]*.

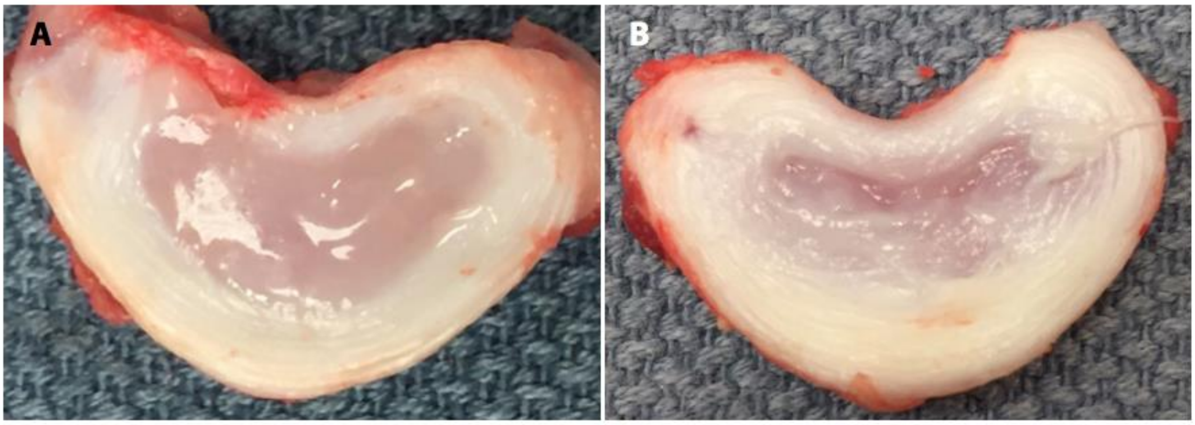

Supplementary Figure 1. Intervertebral disc degeneration following injury. Microphotographs showing a healthy disc (**A**) compared to a fully degenerated disc (**B**) 10 weeks after injury.

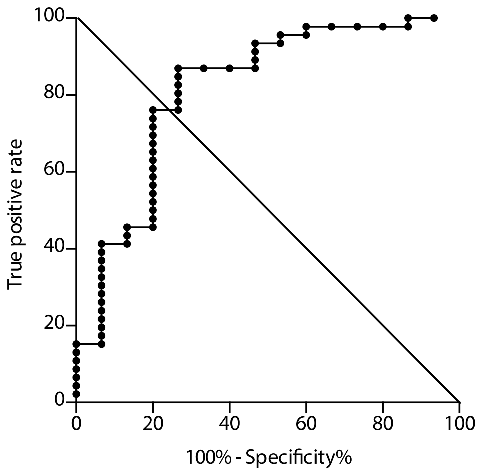

Supplementary Figure 2. ROC curve analysis of qCEST signaling. Receiver operating characteristic (ROC) curve of qCEST measurements for the detection of degenerating IVDs (area under the curve (AUC) = 0.813, p = 0.0003).

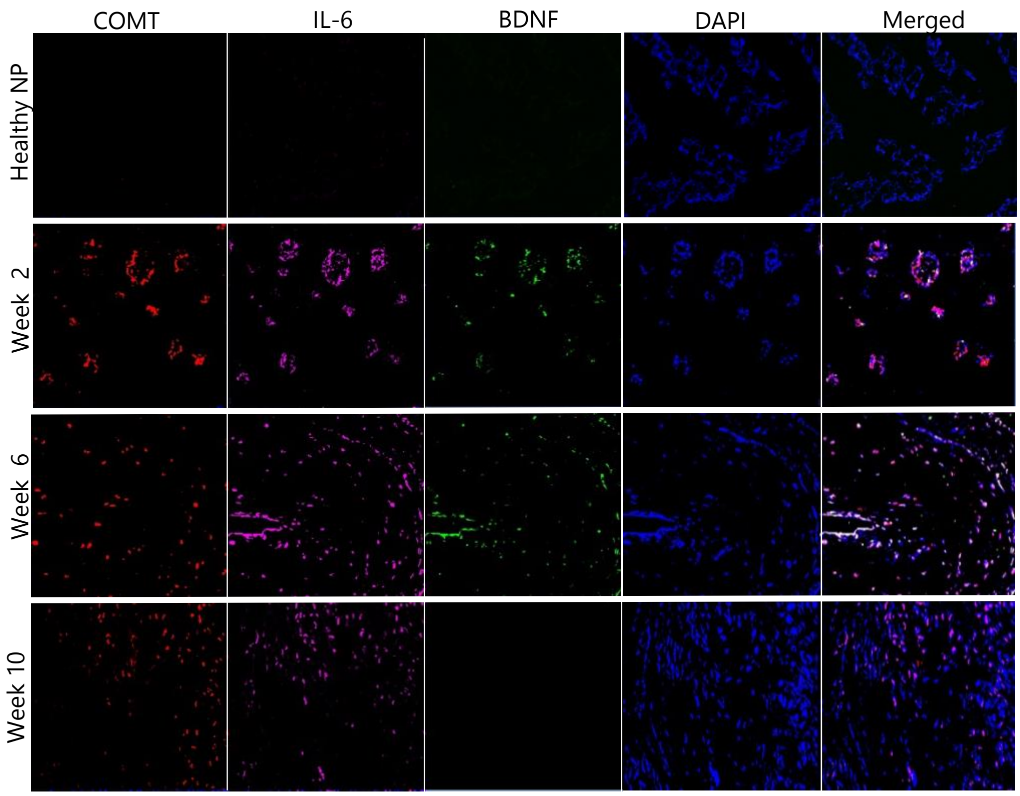

Supplementary Figure 3. Immunofluorescence of inflammation-related marker upregulation. A series of slides of IVDs obtained 2, 6, and 10 weeks after intradiscal puncture, which were immunostained against COMT, IL-6, and BDNF, and counterstained with DAPI. Merged panels of the different stains are presented in the right column. (NP = nucleus pulposus, COMT = catechol-*O*-methyltransferase, BDNF = brain-derived neurotrophic factor).

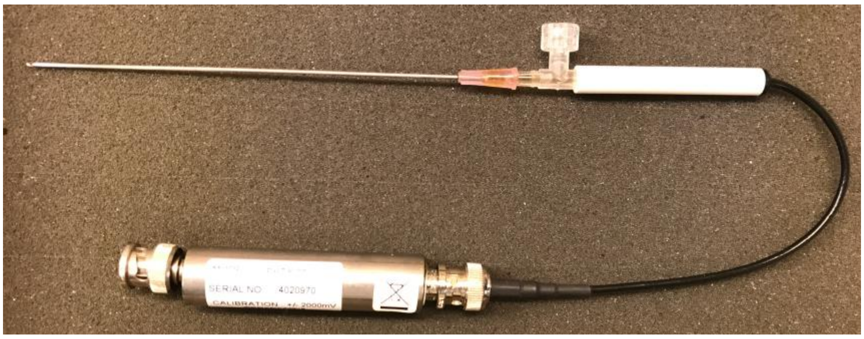

Supplementary Figure 4. Custom-made tissue pH probe. A needle-shaped tissue pH probe was used to measure pH within the IVDs following animal sacrifice.

**Antigen**	**Primary Antibody**	**Secondary Antibody***
BDKRB1	Anti-BDKRB1 antibody TA317572 (OriGene Technologies Inc.)	Donkey Anti-Rabbit Alexa Fluor 488 AffiniPure (cat# 711-545-152)
BDNF	Anti-BDNF antibody MBS2002795 (MyBioSource)	
COMT	Anti-COMT antibody LS-B4343 (LifeSpan Biosciences Inc.).	Donkey Anti-Goat IgG (H+L) Alexa Flour 647 (cat# 705-605-003)
IL-6	Anti-IL6 antibody MAB686 (R&D Systems)	Donkey Anti-Mouse IgG (H+L) ML Rhodamine-TRITC (cat# 715-025-150)
CGRP	Anti-CGRP antibody ab81887 (Abcam)	

Supplementary Table 1. Antibodies used for immunofluorescence throughout the study.

* Purchased from Jackson Immuno Research Laboratories Inc.
